# 2618. Four Respiratory Viruses are Involved in the Majority of Community-Acquired Alveolar Pneumonia (CAAP) Episodes in Children < 5 Years

**DOI:** 10.1093/ofid/ofad500.2231

**Published:** 2023-11-27

**Authors:** Ron Dagan, Bart Adriaan van der Beek, David Greenberg, Shalom Ben-Shimol, Ayelet Keren-Naos, Daniel M Weinberger, Dana Danino

**Affiliations:** Ben-Gurion University of the Negev, Beer Sheva, HaDarom, Israel; Ben-Gurion University of the Negev, Beer Sheva, HaDarom, Israel; Soroka University Medical Center, Pediatric Infectious Disease Unit, Beer Sheva, HaDarom, Israel; Soroka University Medical Center, Beer Sheva, HaDarom, Israel; Soroka University Medical Center, Clinical Virology Laboratory, Beer Sheva, HaDarom, Israel; Yale School of Public Health, New Haven, Connecticut; Soroka University Medical Center, Pediatric Infectious Disease Unit, Beer Sheva, HaDarom, Israel

## Abstract

**Background:**

Community-acquired alveolar pneumonia (CAAP) is considered a bacterial disease in most cases. However, multiple studies in young children have shown a high correlation of its incidence with the epidemiological activity of four respiratory viruses (RSV, hMPV, influenza, and parainfluenza [pneumonia-associated viruses; PAV]). This strongly suggests an important role for bacterial-viral coinfections. The current study examines the detection rate of PAV in radiologically-confirmed CAAP in young children.

**Methods:**

Hospitalization for CAAP episodes and viral detection were prospectively studied among all children under 5 years living in southern Israel (the Negev district); over 95% of these children are born and treated at the only medical center in the region. This prospective active surveillance was previously described (Dagan, EBioMedicine, 90:104493, 2023). Viral activity was defined by all PAV-positive tests in children under 5 years in the community. Nasal samples of children with CAAP were obtained for virus detection by PCR within 48 hours from admission. Only episodes tested for all four PAVs were included. The monthly incidence of PAV-positive CAAP was calculated by extrapolating to all-CAAP episodes, in each month.

**Results:**

During the study period, 2,592 CAAP hospitalizations occurred. Specimens for all four PAVs were obtained in 1,851 (71.4%) CAAP episodes (< 12 months, 1,009; 12-23 months, 443; 24-59 months, 399) (**Table**). Overall, 59% of all episodes were positive for one or more PAVs, 64% of which were RSV (either as single or mixed detection). The surveillance of PAV activity in the community yielded 9,021 positive specimens for one or more PAVs. The dynamic patterns of both all-cause and PAV-positive CAAP hospitalizations from July 2016 through December 2022 closely resembled that of the PAV activity in the community, including the unusual pattern during the COVID-19 years 2020 through 2022 (**Figure 1**). The PAV-CAAP episodes were the main contributors of the CAAP seasonality (**Figure 2**).

**Figure:**
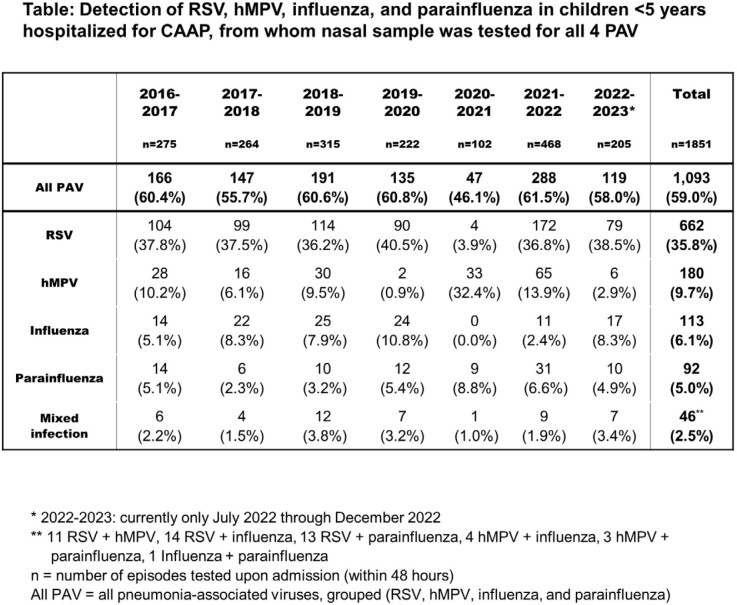

**Figure:**
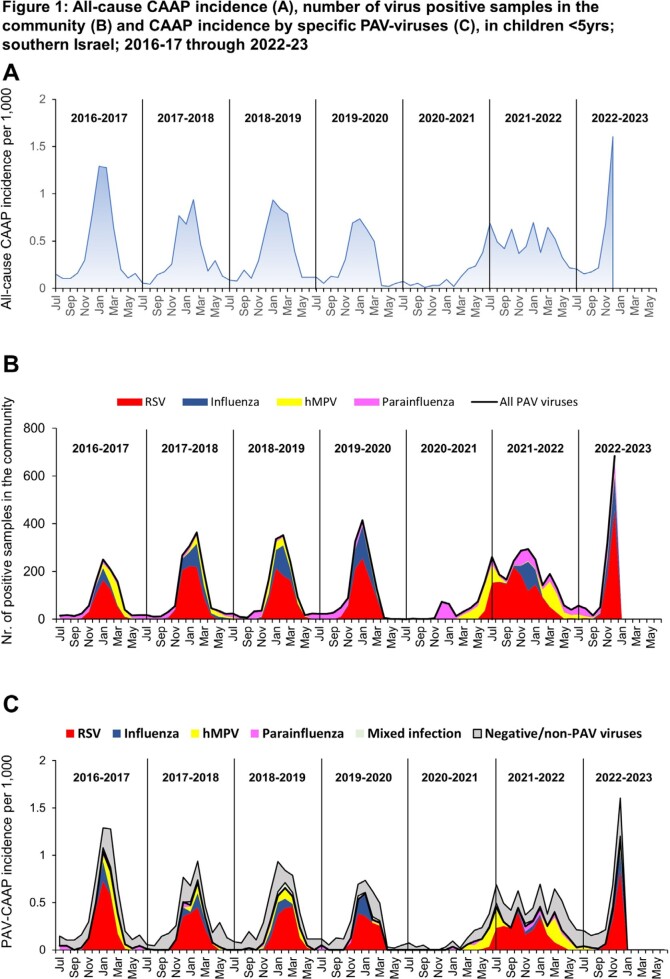

**Figure:**
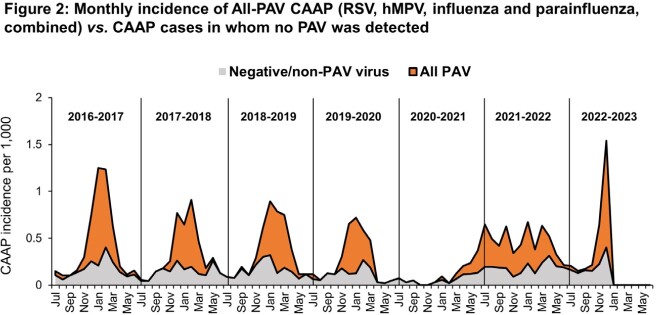

**Conclusion:**

RSV, followed by hMPV, and to a lesser degree influenza and parainfluenza viruses, were the main viruses detected in children under 5 years hospitalized for CAAP. These four viruses were responsible for the seasonal pattern of CAAP hospitalizations.

**Disclosures:**

**Ron Dagan, Professor MD**, GSK: Honoraria|MedIMmune/AstraZeneca: Grant/Research Support|MSD: Advisor/Consultant|MSD: Grant/Research Support|MSD: Honoraria|Pfizer: Advisor/Consultant|Pfizer: Expert Testimony|Pfizer: Grant/Research Support|Pfizer: Honoraria|Sanofi Pasteur: Honoraria **David Greenberg, Professor MD**, GSK: Advisor/Consultant|GSK: Honoraria|MSD: Advisor/Consultant|MSD: Grant/Research Support|MSD: Honoraria|Pfizer: Advisor/Consultant|Pfizer: Honoraria **Shalom Ben-Shimol, Dr. MD**, GSK: Honoraria|MSD: Advisor/Consultant|MSD: Honoraria|Pfizer: Advisor/Consultant|Pfizer: Grant/Research Support|Pfizer: Honoraria **Daniel M. Weinberger, PhD**, GSK: Advisor/Consultant|Merck: Advisor/Consultant|Merck: Grant/Research Support|Pfizer: Advisor/Consultant|Pfizer: Grant/Research Support **Dana Danino, Dr. MD**, Pfizer: Grant/Research Support

